# Inter-species transmission of *Chlamydophila psittaci* from broiler chickens to ducks, turkeys, pigeons, and quails: an experimental study

**DOI:** 10.3389/fvets.2026.1870801

**Published:** 2026-10-22

**Authors:** Ahmed M. E. Hegazy, Amira M. Morsy, Heba M. Salem, Jawza A. Almutairi, Hadil Alkathiry, Yasser S. Mostafa, Ibrahim Mufadhi M. Alanazi, Suad Hamdan Almasoudi, Khaled A. El-Tarabily, Hala M. N. Tolba

**Affiliations:** 1 Department of Avian and Rabbit Medicine, Faculty of Veterinary Medicine, Zagazig University, Zagazig, Egypt; 2 Reference Laboratory for Quality Control on Poultry Production, Department of Poultry Diseases, Animal Health Research Institute, Arish Branch, Agriculture Research Center (ARC), Arish, Egypt; 3 Department of Poultry Diseases, Faculty of Veterinary Medicine, Cairo University, Giza, Egypt; 4 Department of Diseases of Birds, Rabbits, Fish & their Care & Wildlife, School of Veterinary Medicine, Badr University in Cairo (BUC), Badr City, Cairo, Egypt; 5 Department of Pharmaceutical Sciences, College of Pharmacy, Princess Nourah bint Abdulrahman University, Riyadh, Saudi Arabia; 6 Department of Biology, College of Science, Imam Mohammad Ibn Saud Islamic University (IMSIU), Riyadh, Saudi Arabia; 7 Department of Biology, College of Science, King Khalid University, Abha, Saudi Arabia; 8 Department of Pharmacology and Toxicology, Faculty of Medicine, Umm Al-Qura University, Al-Abidiyah, Makkah, Saudi Arabia; 9 Department of Biology, College of Sciences, Umm Al-Qura University, Makkah, Saudi Arabia; 10 Department of Biology, College of Science, United Arab Emirates University, Al Ain, United Arab Emirates

**Keywords:** avian chlamydiosis, bacterial shedding, cross-species transmission, histopathology, psittacosis, qRT-PCR

## Abstract

**Introduction:**

Avian chlamydiosis (psittacosis), caused by *Chlamydia psittaci*, is a contagious bacterial disease of birds with significant economic and zoonotic importance. The infection spreads among avian species and to humans through contaminated secretions, feces, fomites, and close contact, posing challenges to poultry health and public health.

**Methods:**

In the present study, at 1 day old, 90 birds (two chicken groups [one challenged positive control and the other kept unchallenged negative control], ducks, turkeys, pigeons, and quails) were divided into six equal groups (15 birds/group [species]). The birds were reared in a controlled environment on a deep-litter system, with conditions adjusted to their requirements. On the 7th day of age, the first broiler group (G1) was inoculated in both nostrils with 0.2 ml of *C. psittaci* at 10^6^ inclusion-forming units (IFU) ml^−1^. The other four groups of avian species, other than chickens, remained uninfected. When clinical signs appeared in broiler chicks, three infected chicks were transferred to each of the four experimental groups, while the second chicken remained in an unchallenged negative control group.

**Results:**

Clinical signs appeared within 3–5 days post-contact. Turkeys showed the highest overall clinical susceptibility, where depression was observed in 14 of 15 birds (93.3%) and respiratory signs in 13 of 15 birds (86.7%), compared with 6 of 15 birds (40%) in chickens. Ducks, pigeons, and quails showed the lowest susceptibility, with only 5 of 15 birds (33.3%) affected. Postmortem (PM) lesions followed a similar pattern, with turkeys showing the most severe pathological changes. Histopathological examination confirmed inflammatory and degenerative alterations in several organs, particularly in turkeys and ducks. Molecular detection using quantitative real-time polymerase chain reaction (qRT-PCR) confirmed bacterial shedding.

**Discussion:**

The current findings indicate that turkeys were the most susceptible species to *C. psittaci* infection, while chickens showed moderate susceptibility, with responses relatively comparable to ducks, pigeons, and quail, which showed the lowest percentage. Notably, turkeys were the first species to develop clinical signs and exhibited the most severe pathological lesions among the avian species examined. Therefore, it is advisable to enhance biosecurity protocols on mixed-species poultry farms, prioritize surveillance of turkeys given their heightened vulnerability, implement regular molecular monitoring to detect infections early, and segregate avian species during outbreaks to mitigate transmission.

## Introduction

*Chlamydophila psittaci* is an obligate intracellular Gram-negative bacterium belonging to the family Chlamydiaceae (1, 2). It has a unique biphasic developmental cycle consisting of infectious, metabolically inactive elementary bodies (EBs) and non-infectious, metabolically active reticulate bodies (RBs) (3). EBs enable survival outside host cells and initiate infection by attaching to and entering epithelial cells of the respiratory and gastrointestinal tracts (4).

Once internalized, EBs transform into RBs, which replicate within a membrane-bound vacuole known as an inclusion, then reorganize back into EBs for release and dissemination of infection (5). The bacterium lacks peptidoglycan but possesses a major outer membrane protein (MOMP) that plays a critical role in antigenicity and host immune recognition (6). Several genotypes of *C. psittaci* have been identified, showing variable host specificity, pathogenicity, and zoonotic potential, making it an essential pathogen in both avian and human medicine (7, 8).

Avian chlamydiosis (psittacosis) is an infectious disease of birds caused by *C. psittaci*, characterized by its wide host range and zoonotic potential (9, 10). The zoonotic nature of *C. psittaci* underscores its significance within the One Health framework, as it poses occupational health risks to poultry workers, veterinarians, bird handlers, and others in close contact with infected birds. These risks highlight the need for integrated surveillance and control strategies that connect animal, human, and environmental health sectors to effectively prevent and manage disease transmission (11).

The pathogen is transmitted primarily by inhalation of aerosolized droppings or respiratory secretions and can persist in the environment for extended periods (12). Infected birds may also show nonspecific clinical signs, including respiratory distress, conjunctivitis, diarrhea, and reduced productivity. Subclinical infections are also widespread, facilitating silent spread within flocks (13).

Notably, because of its significant impact on poultry health and productivity and its potential to cause psittacosis in humans, avian chlamydiosis is both an economic and public health concern (13). Additionally, cross-species transmission of avian chlamydiosis occurs when *C. psittaci* is transmitted between avian species or from birds to mammals, including humans (14, 15). Moreover, human-to-human transmission has just been verified (16).

Additionally, the pathogen is primarily shed in respiratory secretions and feces, where it can persist in the environment as infectious EBs (17). Close contact, shared housing, contaminated feed, water, or fomites facilitate interspecies transmission among domestic poultry, wild birds, and pet birds (18, 19). Notably, the disease's zoonotic nature facilitates transmission to humans, particularly among individuals with occupational exposure, such as poultry workers, veterinarians, and bird handlers (20, 21). Such cross-species transmission not only complicates disease control in avian populations but also poses a significant public health risk (22).

Avian chlamydiosis has variable clinical manifestations, morbidity, and mortality depending on the bird species, age, and immune status (6, 9). In psittacine birds, signs often include lethargy, anorexia, conjunctivitis, nasal discharge, greenish diarrhea, and respiratory distress, with morbidity reaching high levels, while mortality may vary from low to severe in acute cases (23, 24).

In domestic poultry, such as turkeys and ducks, outbreaks are associated with respiratory signs, reduced egg production, weight loss, and, occasionally, sudden death (9). Morbidity can be significant, though mortality is often moderate unless secondary infections are present (25). Postmortem (PM) findings commonly include air sacculitis, fibrinous pericarditis, hepatosplenomegaly with small grayish necrotic foci, and serofibrinous exudates in body cavities (26).

In turkeys, quails, pigeons, and other wild birds, the lesions are often nonspecific but may include emaciation, an enlarged, mottled liver, and serositis (27, 28). These diverse clinical and pathological presentations complicate diagnosis and highlight the importance of laboratory confirmation (29).

Accordingly, the current study aimed to investigate the interspecies transmission dynamics and comparative susceptibility of different avian species (chickens, ducks, turkeys, pigeons, and quails) to *C. psittaci* infection under controlled experimental conditions, with emphasis on clinical outcomes, bacterial shedding, and histopathological changes.

## Materials and methods

### Ethical approval

The present study was conducted in accordance with the committee's guidelines and received ethical approval (ZU-IACUC/2/F/41/2023) from the Zagazig University Institutional Animal Care and Use Committee, Faculty of Veterinary Medicine, Zagazig University, Zagazig, Egypt.

### Bacterial inoculum

The *C. psittaci* field isolate was originally isolated from naturally infected broiler Cobb chickens in Sharqia governorate, Egypt, in 2014. The isolate was identified serologically by complement fixation test (CFT), molecularly by quantitative real-time polymerase chain reaction (qRT-PCR), and by immunofluorescence assay for *C. psittaci* antigen detection as reported by Tolba et al. (30).

The isolate was passed 3 times in the yolk sac (YS) of specific pathogen free (SPF) eggs. It was stored at −80°C as a 20% YS suspension in sucrose phosphate glutamate (SPG) until use (31).

### Experimental design and animal allocation

All birds were randomly assigned to experimental and control groups to minimize selection bias. Investigators responsible for clinical scoring, histopathological assessment, and laboratory analyses were blinded to group assignments to ensure objective evaluation.

### Housing and separation protocols

Ninety (one-day-old) birds from five avian species (chickens, ducks, turkeys, pigeons, and quail) were used in the current study. Birds were randomly divided into six experimental groups, with 15 birds assigned to each group. The groups were designated as follows: G1 (chickens, challenged positive control), G2 (chickens, unchallenged negative control), G3 (ducks), G4 (turkeys), G5 (pigeons), and G6 (quail).

All birds were reared under controlled environmental conditions on a straw-bedded deep-litter system with species-specific management practices, including appropriate temperature, humidity, and lighting regimens that were adjusted to meet species requirements, and had *ad libitum* access to feed and water.

### Sample collection and bacterial detection

During the 1st week, oropharyngeal and cloacal swabs from three randomly selected birds of each species were tested using qRT-PCR to confirm the absence of *C. psittaci*.

At 7 days of age, chickens (G1) were intranasally inoculated with 0.2 mL of *C. psittaci* at 10^6^ inclusion-forming units (IFU) ml^−1^, while the remaining four groups from avian species other than chickens were uninfected. Once clinical signs appeared in chickens, including ruffled feathers, lethargy, depression, diarrhea, nasal discharge, and conjunctivitis, three infected birds were introduced into each of the other four groups to allow horizontal transmission.

In contrast, the second chicken group remained in an unchallenged negative control group. Clinical signs and PM lesions were recorded throughout the experiment until 35 days of age. Oropharyngeal swabs were collected at 7, 10, and 14 days post-infection (dpi) from all groups for qRT-PCR detection of bacterial shedding.

### Histopathological techniques and lesion scoring

Tissue samples were collected from 3 birds from each bird species after humane and ethical slaughter of the birds, including the heart, liver, kidney, lung, and spleen. Samples were fixed, processed, and stained with hematoxylin and eosin. Lesions were scored according to severity and extent using standardized criteria.

For histopathology, samples of the heart, liver, kidney, lung, and spleen were collected from chickens, turkeys, ducks, pigeons, and quails at 7, 10, and 14 dpi. Tissues were fixed in 10% neutral-buffered formalin for 24 h, dehydrated in ascending grades of ethanol, cleared in xylene, and embedded in paraffin wax. Paraffin blocks were sectioned at 5 μm thickness using a microtome (Leica RM 2,155, London, UK), and sections were stained with hematoxylin and eosin following standard protocols (32). Stained slides were examined under a Leica^®^ microscope equipped with an AmScope^®^ digital camera.

Histopathological changes were graded semi-quantitatively in five randomly selected microscopic fields per tissue according to Gibson-Corley et al. (33): (0) no alterations, (1) mild, (2) moderate, and (3) severe alterations. Lesion scores were tabulated separately for each avian species.

Lesion scoring categories were tailored to each species to capture the full spectrum of pathological changes. Consequently, direct cross-species comparisons should be interpreted with caution, and the species-specific nature of the scoring criteria is acknowledged as a limitation.

### Molecular diagnosis

DNA was isolated from oropharyngeal swabs following the protocol of Magnino et al. (34) targeting the 23S rRNA gene of *C. psittaci* using QIAamp DNA Mini Kit (Qiagen, Hilden, Germany). The extracted DNA was dissolved in 50 μl of Qiagen elution buffer and preserved frozen at −20°C until analysis.

Positive and/or negative controls (35) were represented by field samples that were previously confirmed to be positive or negative by PCR for the related gene in a reference laboratory for veterinary quality control on poultry production, the Animal Health Research Institute. The 23SrRNA region of the extracted DNA was amplified by qRT-PCR using the primers: CPsitt-F (5′0-TTACCACATGCATGGAAGCG- 3′0) and CPsitt-R (5′0- CGTTCGATTTCTGCTTTTTCTCTA - 3′0) (36).

The PCR was performed using a 20 μl reaction volume containing iQ Multiplex PowerMix, bovine serum albumin (0.5 μg μl^−1^), primers (0.3 μm each), probes (0.2 μm each), and 1–2 μl of template DNA. All samples were analyzed in duplicate or triplicate (36) (Table 1).

**Table 1:** Oligonucleotide primers and probes for *Chlamydophila psittaci*.

Agent	Primer/probe sequence 5^′^-3^′^	Gene	Reference
*Chlamydophila psittaci*	CPsitt-F (5′0-TTACCACATGCATGGAAGCG- 3′0)	23S rRNA	(36)
	CPsitt-R (5′0- CGTTCGATTTCTGCTTTTTCTCTA - 3′0)		

The reactions were carried out in a thermal cycler (Mastercycler gradient; Eppendorf, Hamburg, Germany) with an initial denaturation step for 3 min at 95°C. An amplification step was performed for 45 cycles: denaturation for 15 s at 95°C, annealing for 1 min at 60°C, extension for 1 min at 72°C, and a final extension for 7 min at 72°C. The PCR products were run on a 1.5% agarose gel and visualized using ethidium bromide staining under UV light. The DNA molecular weight marker served as a size standard, and a sample was deemed positive when the amplification product specific to *C. psittaci* was detected at 151 bp (36).

### Statistical analysis

Statistical analysis was performed using GraphPad Prism (version 10.0; GraphPad Software, San Diego, CA, USA). Gross pathological lesions and clinical signs were analyzed as categorical variables. For each lesion and clinical sign, prevalence was summarized as the number and percentage of affected birds within each avian species.

Associations between bird species and lesion or clinical sign occurrence were evaluated using Pearson's Chi-square test of independence. Where expected cell frequencies were insufficient for reliable Chi-square approximation, Fisher's exact test was used whenever computationally feasible. Standardized Pearson residuals were examined to identify the cell(s) contributing most to significant associations. Statistical significance was defined as *P* < 0.05. (37).

## Results

### Mortality, clinical, and PM scoring

The negative control chicken group showed neither mortality nor clinical signs throughout the observation period. During the 35-day experimental period, no mortality was recorded in any experimental group.

The distribution of clinical manifestations demonstrated significant species-dependent variability (Table 2). Turkeys exhibited the highest clinical morbidity across all signs, most notably in depression (93.3%) and respiratory distress (86.7%), both of which were significantly associated with species type (*P* = 0.002 and *P* = 0.01, respectively).

**Table 2:** Distribution of clinical manifestations among bird species.

Species	Depression *n* (%)	Respiratory signs *n* (%)	Diarrhea *n* (%)
Chicken	6 (40.0)	6 (40.0)	5 (33.3)
Duck	6 (40.0)	5 (33.3)	10 (66.7)
Turkey	14 (93.3)	13 (86.7)	10 (66.7)
Quail	4 (26.7)	5 (33.3)	4 (26.7)
Pigeon	6 (40.0)	5 (33.3)	10 (66.7)
*P-*value	0.002	0.01	0.04

*n* = number of birds.

While diarrhea was also significantly associated with species (*P* = 0.04), it showed a broader distribution, with ducks, pigeons, and turkeys all displaying higher prevalence (66.7%) compared to chickens and quails. These findings indicate that clinical expression is not uniform among avian species, with turkeys demonstrating a marked predisposition to severe clinical illness. This non-random clustering of clinical signs suggests that species-specific physiological responses or varying exposure dynamics strongly influence the symptomatic presentation of the infection, as shown in Figures 1, 2.

**Figure 1:**
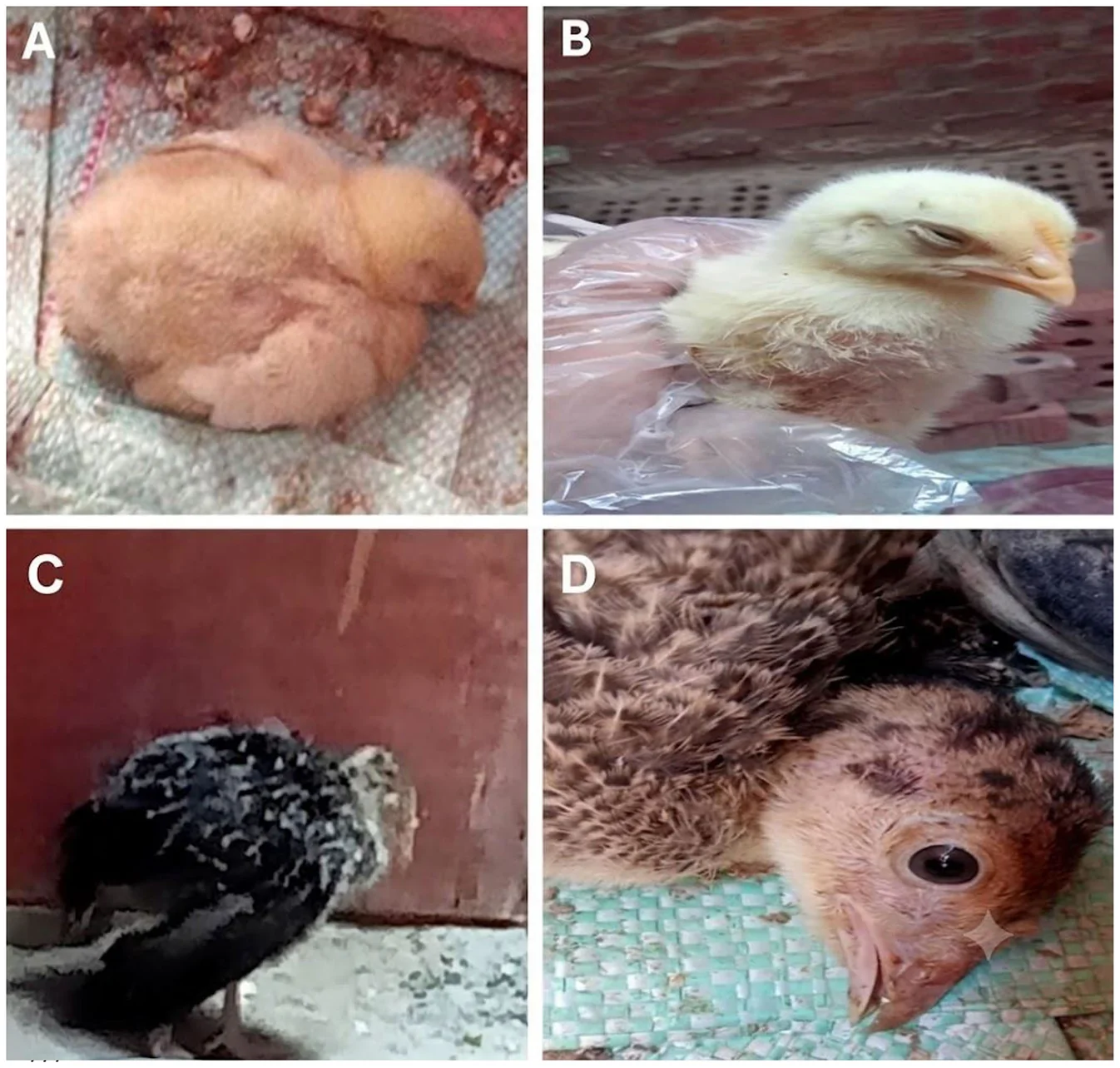
Clinical signs in different experimental groups [chickens experimentally infected with *Chlamydophila psittaci* and contacted birds (turkeys and quails)] showing **(A)**: chicks with ruffled feather, sleepy appearance with depression in chicks at 7th dpi; **(B)**: chicks with respiratory signs (nasal discharge) and conjunctivitis in chicks at 10th dpi; **(C)**: turkeys with ruffled feather with depression in turkeys at 7th dpi; and **(D)**: turkeys with respiratory signs (nasal discharge), open beak and unilateral sinusitis in turkeys at 14th dpi. dpi, days post-infection.

**Figure 2:**
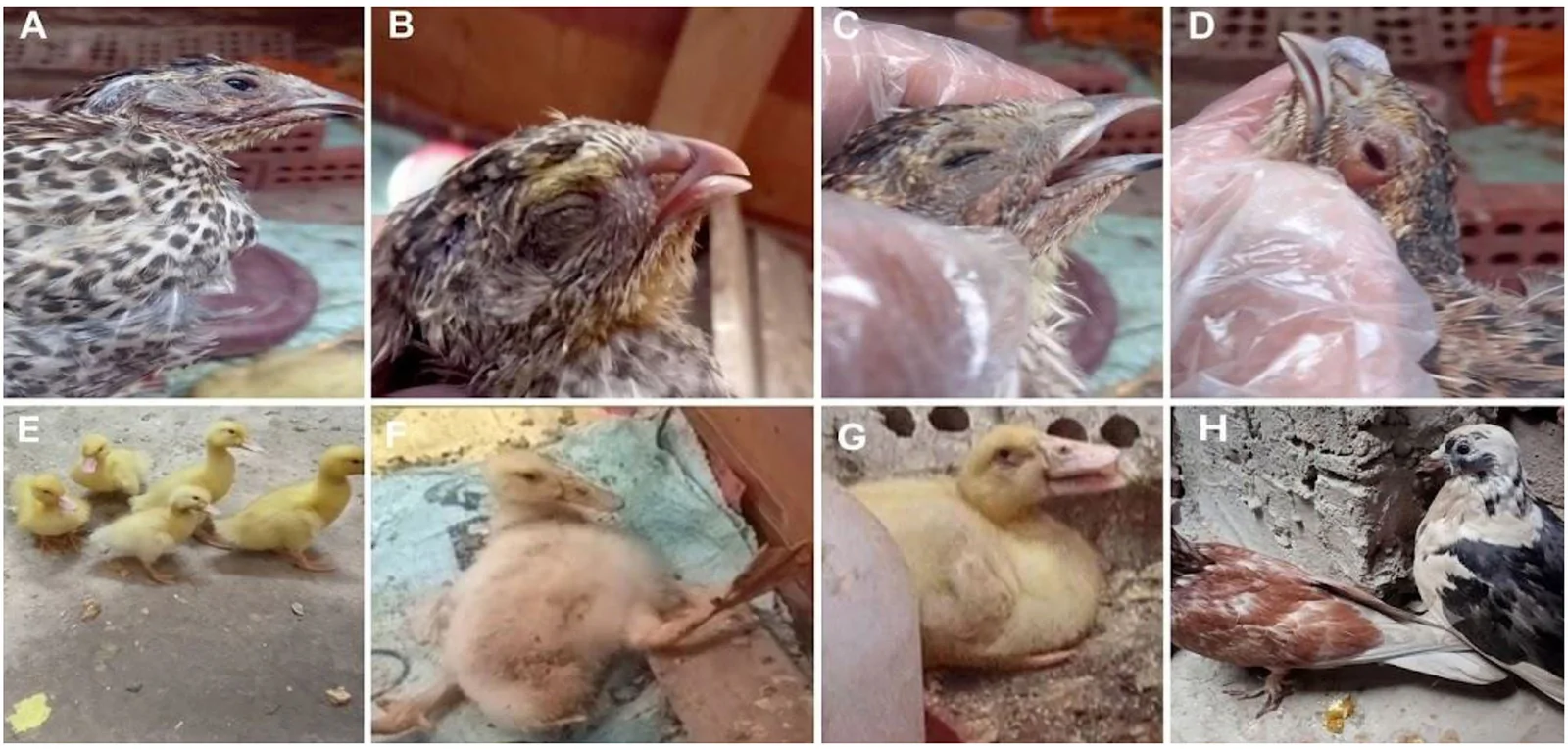
Clinical signs in different experimental birds groups contacted with chickens experimentally infected with *Chlamydophila psittaci* showing **(A)**: quails with unilateral conjunctivitis at 10th dpi; **(B)**: quails with complete closure of eye with conjunctivitis, nasal and ocular discharge at 7th dpi; **(C)**: quails with mouth breathing at 14th dpi; **(D)**: quails with edema eyelids and conjunctivitis at 14th dpi; **(E)**: ducks with yellow to greenish diarrhea and depression at 107th dpi; **(F)**: ducks with nervous signs (paddling) at 14th dpi; **(G)**: ducks with gasping of air at 14th dpi; and **(H)**: Baladi pigeons with greenish brownishw diarrhea with depression at 10th dpi. dpi, days post-infection.

This disproportionate impact is visually emphasized in Supplementary Figure S1 (Panels A–C), where the turkey cohort consistently accounts for the largest share of clinical presentations, representing 39% of all depression cases and 38% of respiratory observations. While diarrhea also showed a significant association with species (*P* = 0.04), it displayed a more uniform distribution, with ducks, turkeys, and pigeons contributing roughly equal shares (25%−26% each) to the overall morbidity.

These findings indicate that clinical expression is non-uniform among avian species; the marked clustering of severe symptoms in turkeys suggests a heightened species-specific predisposition, warranting further investigation into whether these birds act as primary reservoirs or possess unique physiological vulnerabilities to the causal agent.

PM examination of infected birds revealed a spectrum of gross pathological lesions affecting multiple organ systems. The most frequently observed lesions across the different avian species included hepatomegaly with marked congestion, renal congestion, pericarditis, air sacculitis, pulmonary congestion, splenomegaly, and enteritis. The distribution and severity of these lesions exhibited notable species-dependent variation.

Species-specific differences were highly significant across all evaluated pathological lesions (Pearson's χ^2^; *P* < 0.001). Turkeys showed the highest susceptibility, with 100% prevalence of liver lesions, spleen lesions, and air sacculitis, and a 66.7% incidence of enteritis. In contrast, chickens, ducks, quails, and pigeons displayed markedly lower and more variable lesion frequencies, with air sacculitis being the predominant lesion in chickens and ducks (66.7%), while splenic involvement was least frequent in quails (20%) as shown in Supplementary Figure S1.

Standardized residual analyses indicate that these associations are primarily driven by the disproportionate disease burden in turkeys and the absence of lesions in the negative control group. This is visually corroborated in Panels A–D of Supplementary Figure S2, where turkeys consistently account for the largest share of organ lesion distribution, reaching up to 41% of total observed splenic lesions. Collectively, while the pattern of PM lesion occurrence is broadly similar across species, the distinct clustering of severe pathology in turkeys' points to a heightened species-specific vulnerability, suggesting that turkeys may either act as a primary reservoir or possess a unique physiological susceptibility to the underlying pathogenic factors (Table 3 and Figure 3).

**Table 3:** Distribution of gross pathological lesions among different avian species.

Species	Liver lesion *n* (%)	Spleen lesion *n* (%)	Air sacculitis *n* (%)	Enteritis *n* (%)
Negative	0 (0.0)	0 (0.0)	0 (0.0)	0 (0.0)
Chicken	6 (40.0)	6 (40.0)	10 (66.7)	5 (33.3)
Duck	6 (40.0)	6 (40.0)	10 (66.7)	10 (66.7)
Turkey	15 (100.0)	15 (100.0)	15 (100.0)	10 (66.7)
Quail	6 (40.0)	3 (20.0)	8 (53.3)	8 (53.3)
Pigeon	6 (40.0)	6 (40.0)	5 (33.3)	10 (66.7)
*P*-value	*P* < 0.001	*P* < 0.001	*P* < 0.001	*P* < 0.001

Values are presented as the number of birds exhibiting each lesion [*n* (%)]. Percentages were calculated within each bird species using the total number of examined birds of that species as the denominator (i.e., Percentage = (number of birds with the lesion ÷ total number of birds in that species) × 100). Associations between bird species and each pathological lesion were evaluated using Pearson's Chi–square test.

**Figure 3:**
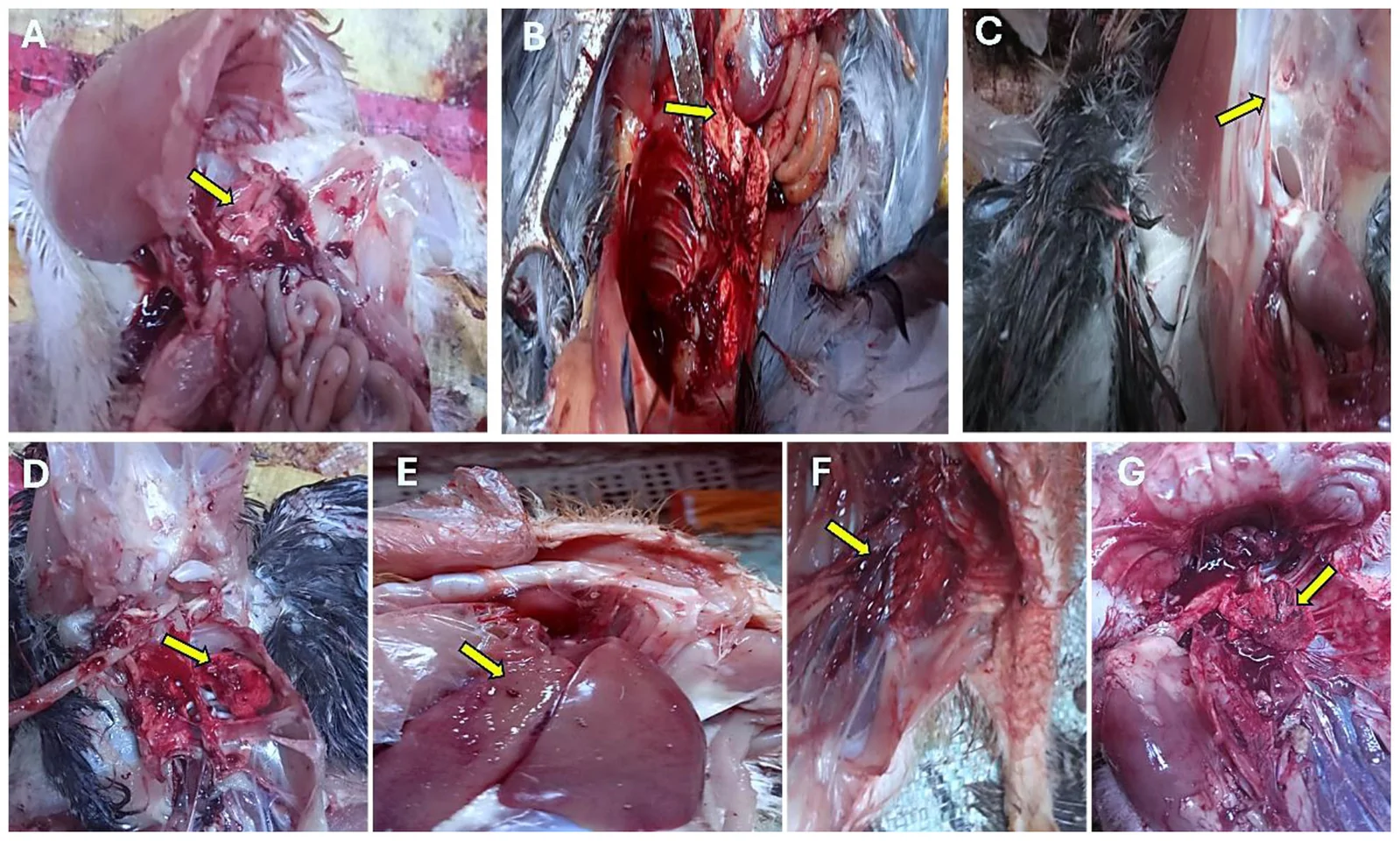
Postmortem lesions among different experimental groups [infected chickens with *Chlamydophila psittaci*, and contacted birds (ducks, turkeys, and quails)] showing **(A)**: chickens with congested lung at 10th dpi; **(B)**: turkeys with congested lung at 7th dpi; **(C)**: quails with mild air sacculitis at 14th dpi; **(D)**: quails with congested lung at 7th dpi; **(E)**: ducks with enlarged liver at 7th dpi; **(F)**: ducks with congested kidney at 10th dpi; and **(G)**: ducks with congested lung at 14th dpi. dpi, days post-infection.

### Histopathological studies

Histopathological examination revealed species- and time-dependent tissue alterations following infection, with lesion severity generally increasing and reaching its maximum between 7 and 10 dpi, followed by substantial recovery by 14 dpi. Lesion nature and severity varied considerably among bird species and examined organs.

Negative control birds of all examined species exhibited normal histological architecture in the heart, liver, kidney, lung, and spleen, with no detectable pathological lesions throughout the study period (Supplementary Figure S3). Histopathological lesion severity scores presented in Tables 4–8, Figures 4–8, and Supplementary Figures S4–S8 further support these observations and demonstrate the progressive development and subsequent resolution of tissue damage over the experimental period.

**Table 4:** Lesion score of the severity extent in the heart, liver, kidney, lung, and spleen tissues among experimentally infected chickens with *Chlamydophila psittaci*.

Organ	Lesions	7th dpi	10th dpi	14th dpi
Heart	Thickened pericardium	1	0	0
	Degenerated cardiomyocytes	1	1	0
	Dilated interstitial blood vessels	2	0	0
Liver	Degenerated hepatocytes	1	1	0
	Interstitial inflammatory cells	2	1	0
	Congested hepatic blood vessels	2	1	0
Kidney	Degenerated tubules	0	2	1
	Necrotic tubules	0	1	0
	Dilated tubular lumina	0	2	0
	Congestion	0	1	1
Lung	Congestion	1	0	0
	Perivascular edema	1	1	0
	Lymphocytic aggregates	1	1	1
	Spleen	Depleted lymphoid elements	0	0	0

The lesions were graded by estimating the percentage of the section area affected. The lesions score system was as follows: 0 = absence of lesion, 1 = 5–25%, 2 = 26%−50%, and 3 = >50%. dpi, days post-infection.

**Table 5:** Lesion score of the severity extent in the heart, liver, kidney, lung, and spleen tissues among different experimental groups of turkeys contacted with experimentally infected chickens with *Chlamydophila psittaci*.

Organ	Lesions	7th dpi	10th dpi	14th dpi
Heart	Thickened pericardium	1	2	0
	Degenerated cardiomyocytes	1	2	0
	Inflammatory cells infiltrate	1	1	0
Liver	Degenerated hepatocytes	3	1	0
	Interstitial inflammatory cells	2	2	0
	Congested hepatic blood vessels	1	2	0
Kidney	Degenerated tubules	2	1	1
	Necrotic tubules	2	1	0
	Congestion	1	2	0
Lung	Congestion	1	1	0
	Perivascular edema	2	1	0
	Lymphocytic aggregates	1	2	2

The lesions were graded by estimating the percentage area affected in the entire section. The lesions score system was as follows: 0 = absence of lesion, 1 = 5–25%, 2 = 26%−50%, and 3 = >50%. dpi, days post-infection.

**Table 6:** Lesion score of the severity extent in the heart, liver, kidney, lung, and spleen tissues among different experimental groups of pigeons contacted with experimentally infected chickens with *Chlamydophila psittaci*.

Organ	Lesions	7th dpi	10th dpi	14th dpi
Heart	Thickened pericardium	0	0	0
	Degenerated cardiomyocytes	0	0	0
	Dilated interstitial blood vessels	0	0	0
Liver	Degenerated hepatocytes	2	3	1
	Necrotic areas	2	3	0
	Interstitial inflammatory cells	2	2	1
	Congested hepatic blood vessels	2	3	0
Kidney	Degenerated tubules	1	1	1
	Necrotic tubules	2	1	0
	Dilated tubular lumina	2	0	0
	Interstitial inflammatory cells	2	1	0
	Congestion	1	1	0
Lung	Congestion	1	0	0
	Perivascular edema	2	0	0
	Lymphocytic aggregates	2	1	1

The lesions were scored by assessing the percentage area affected in the entire section. The lesions score system was as follows: 0 = absence of lesion, 1 = 5–25%, 2 = 26%−50%, and 3= >50%. dpi, days post-infection.

**Table 7:** Lesion score of the severity extent in the heart, liver, kidney, lung, and spleen tissues among different experimental groups of quails contacted with experimentally infected chickens with *Chlamydophila psittaci*.

Organ	Lesions	7th dpi	10th dpi	14th dpi
Heart	Thickened pericardium	0	0	0
	Degenerated cardiomyocytes	0	0	0
	Dilated interstitial blood vessels	0	1	0
Liver	Degenerated hepatocytes	0	0	1
	Necrotic areas	0	0	0
	Interstitial inflammatory cells	0	0	0
	Congested hepatic blood vessels	1	1	0
Kidney	Degenerated tubules	0	0	0
	Necrotic tubules	0	0	0
	Dilated tubular lumina	0	0	0
	Interstitial inflammatory cells	0	0	0
	Congestion	0	0	0
Lung	Congestion	0	0	1
	Perivascular edema	1	0	0
	Lymphocytic aggregates	1	0	0

The lesions were scored by assessing the percentage area affected in the entire section. The lesions scoring was as follows: 0 = absence of lesion, 1 = 5%−25%, 2 = 26%−50%, and 3 = >50%. dpi, days post-infection.

**Table 8:** Lesion score of the severity extent in the heart, liver, kidney, lung, and spleen tissues among different experimental groups of ducks contacted with experimentally infected chickens with *Chlamydophila psittaci*.

Organ	Lesions	7th dpi	10th dpi	14th dpi
Heart	Thickened pericardium	0	2	1
	Degenerated cardiomyocytes	0	1	0
	Inflammatory cells infiltration	0	2	1
	Dilated interstitial blood vessels	0	1	0
Liver	Degenerated hepatocytes	2	3	1
	Necrotic areas	0	2	0
	Interstitial inflammatory cells	0	2	0
	Congested hepatic blood vessels	1	2	1
Kidney	Degenerated tubules	0	2	0
	Necrotic tubules	0	2	0
	Dilated tubular lumina	0	1	0
	Interstitial inflammatory cells	0	2	1
	Congestion	0	1	1
Lung	Congestion	0	2	1
	Perivascular edema	0	2	0
	Exudates	0	3	0
	Lymphocytic aggregates	0	2	1

The lesions were scored by assessing the percentage area affected in the entire section. The lesion score system was as follows: 0 = absence of lesion, 1 = 5%−25%, 2 = 26%−50%, and 3 = >50%. dpi, days post-infection.

**Figure 4:**
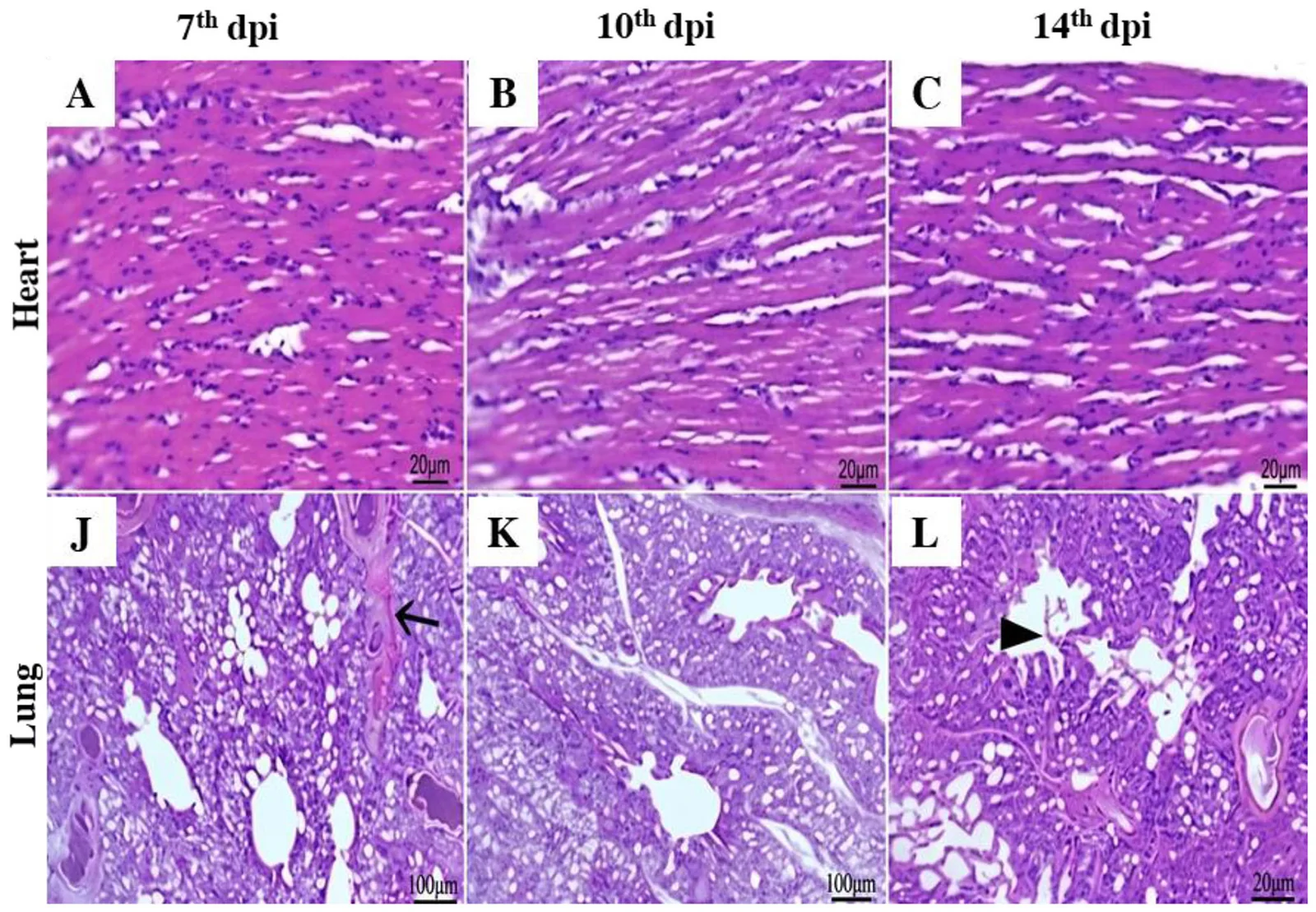
Photomicrograph of hematoxylin and eosin-stained sections from chickens experimentally infected with *Chlamydophila psittaci* showing heart tissues **(A–C)**. **(A)**: apparent normal cardiomyocytes at 7th dpi. **(B)**: apparent normal cardiomyocytes with hyaline degeneration of some myofibers at 10th dpi. **(C)**: normal histological structures of heart muscles at 14th dpi, scale bar (20 μm). Pulmonary tissues **(J–L)**. **(J)**: apparent normal pulmonary tissues with congested blood vessels, and perivascular edema (arrow) at 7th dpi. **(K)**: apparent normal pulmonary configurations at 10th dpi. **(L)**: apparent normal pulmonary architectures with remnant of exudation at some bronchial lumina (arrowhead) at 14th dpi, scale bar (100 μm). dpi, days post-infection.

**Figure 5:**
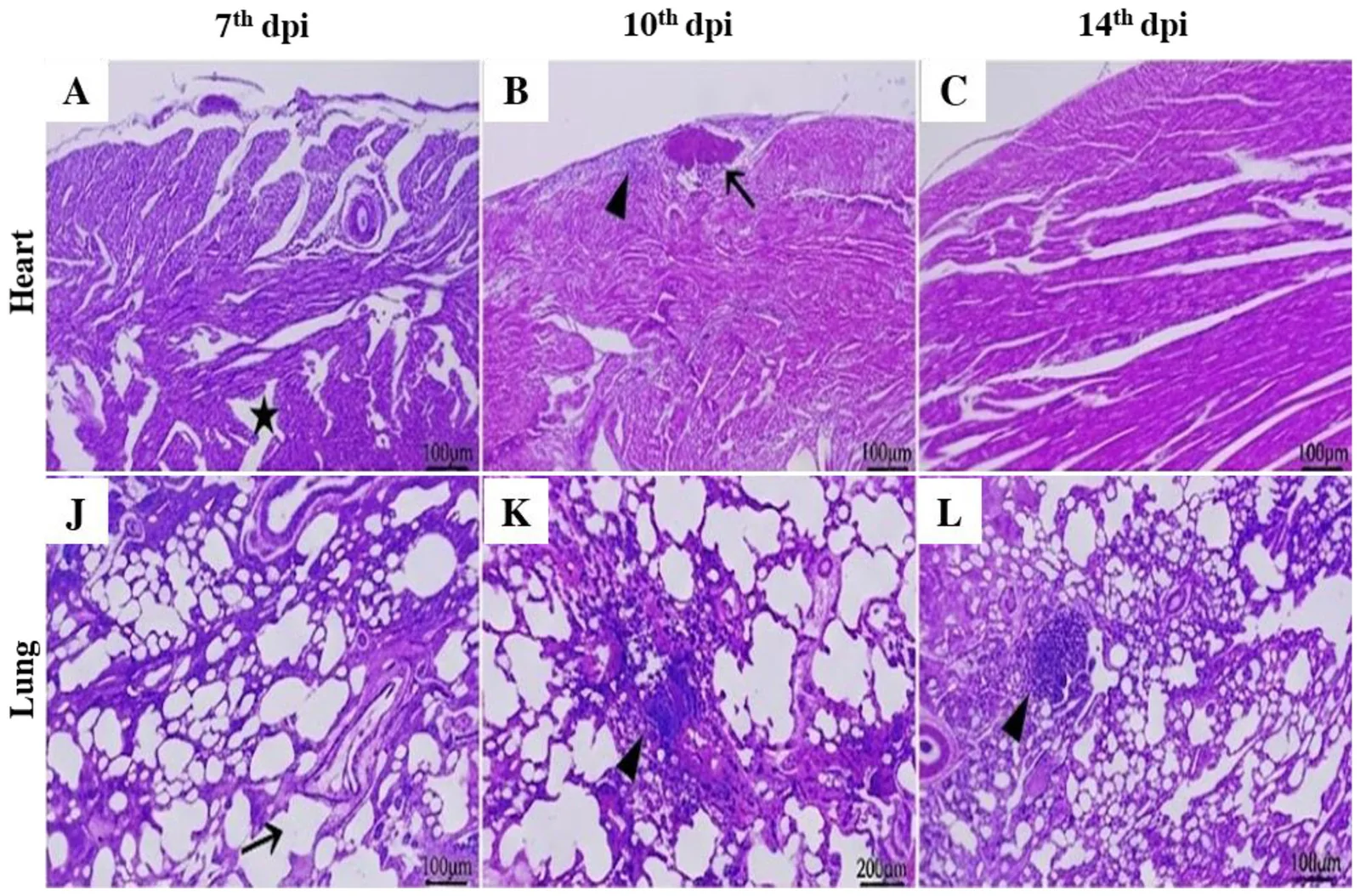
Photomicrograph of hematoxylin and eosin-stained sections from turkeys contacted with chickens experimentally infected with *Chlamydophila psittaci* showing heart tissues **(A–C)**. **(A)**: apparent normal cardiac muscle fibers with mild thickening of pericardium, and mild interstitial edema (star) at 7th dpi. **(B)**: thickened pericardium by congested capillaries (arrow), inflammatory cells (arrowhead) at 10th dpi. **(C)**: normal histological structures of cardiomyocytes and interstitial tissue in the heart tissues at 14th dpi, scale bar (100 μm). Pulmonary tissues **(J–L)**. **(J)**: apparent normal respiratory airways with perivascular edema and hemorrhages (arrow) at 7th dpi. **(K)**: normal histology of pulmonary tissue with focal aggregates of lymphoid elements at peribronchial tissues (arrowhead) at 10th dpi. **(L)**: normal histological structures of bronchi, bronchioles, air vesicles, and vascular tissues with prominent BALT (arrowhead) at 14th dpi. Scale bar (100 μm). dpi, days post-infection.

**Figure 6:**
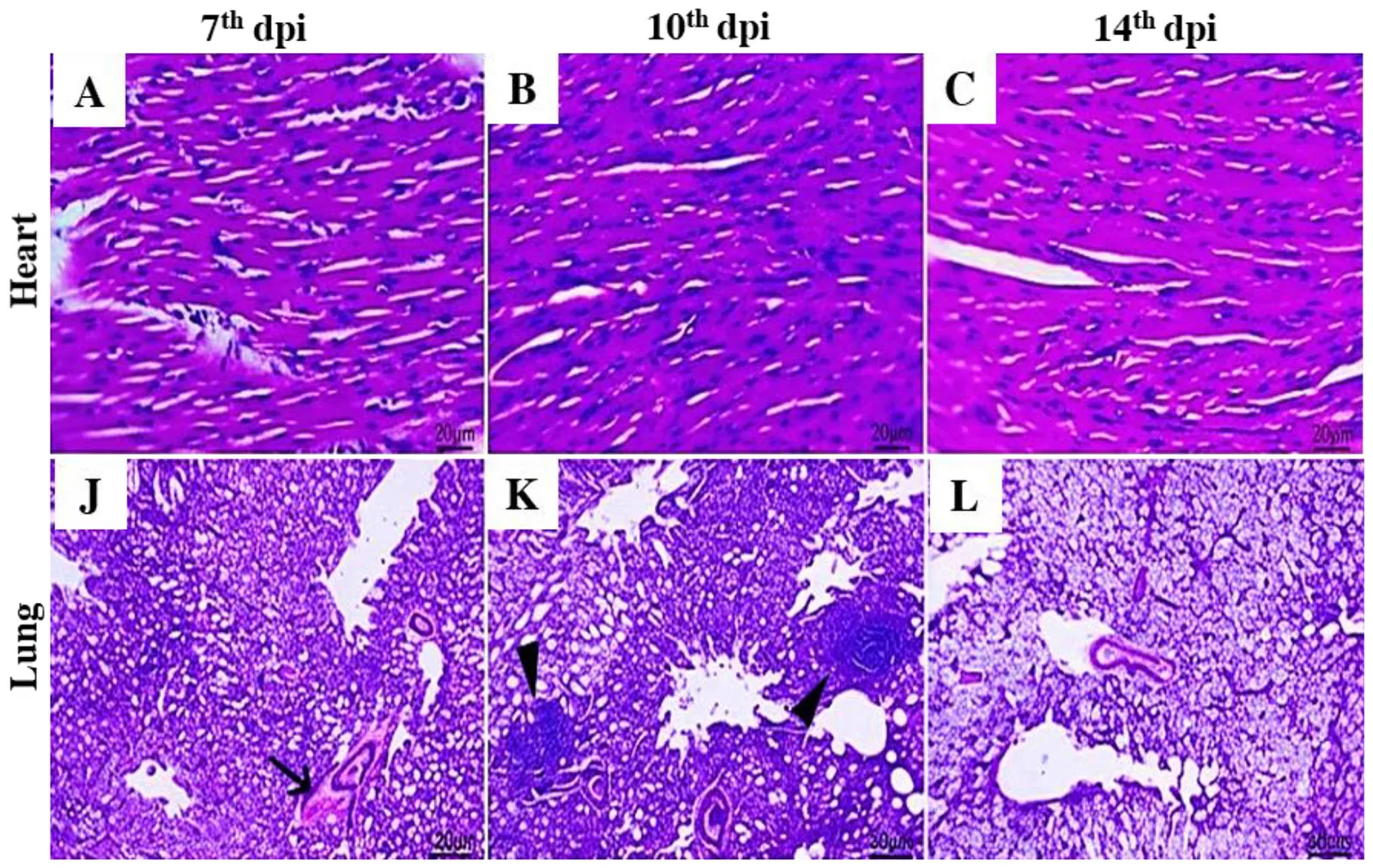
Photomicrograph of hematoxylin and eosin-stained sections from pigeon contacted with chickens experimentally infected with *Chlamydophila psittaci* showing heart tissues **(A–C)**. **(A–C)**: normal histological structures of cardiac muscle fibers and interstitial tissue 7th dpi, and at 14th dpi, scale bar (20 μm). Pulmonary tissues **(J–L)**. **(J)**: apparent normal most respiratory airways with perivascular edema (arrow) and infiltration of some air vesicles by inflammatory cells at 7th dpi. **(K)**: normal histology of pulmonary tissue with prominent peribronchial lymphoid aggregates “BALT” (arrowheads) at 10th dpi. **(L)**: normal histological structures of bronchi, bronchioles, air vesicles, and vascular tissues (arrowhead) at 14th dpi, scale bar (100 μm). dpi, days post-infection.

**Figure 7:**
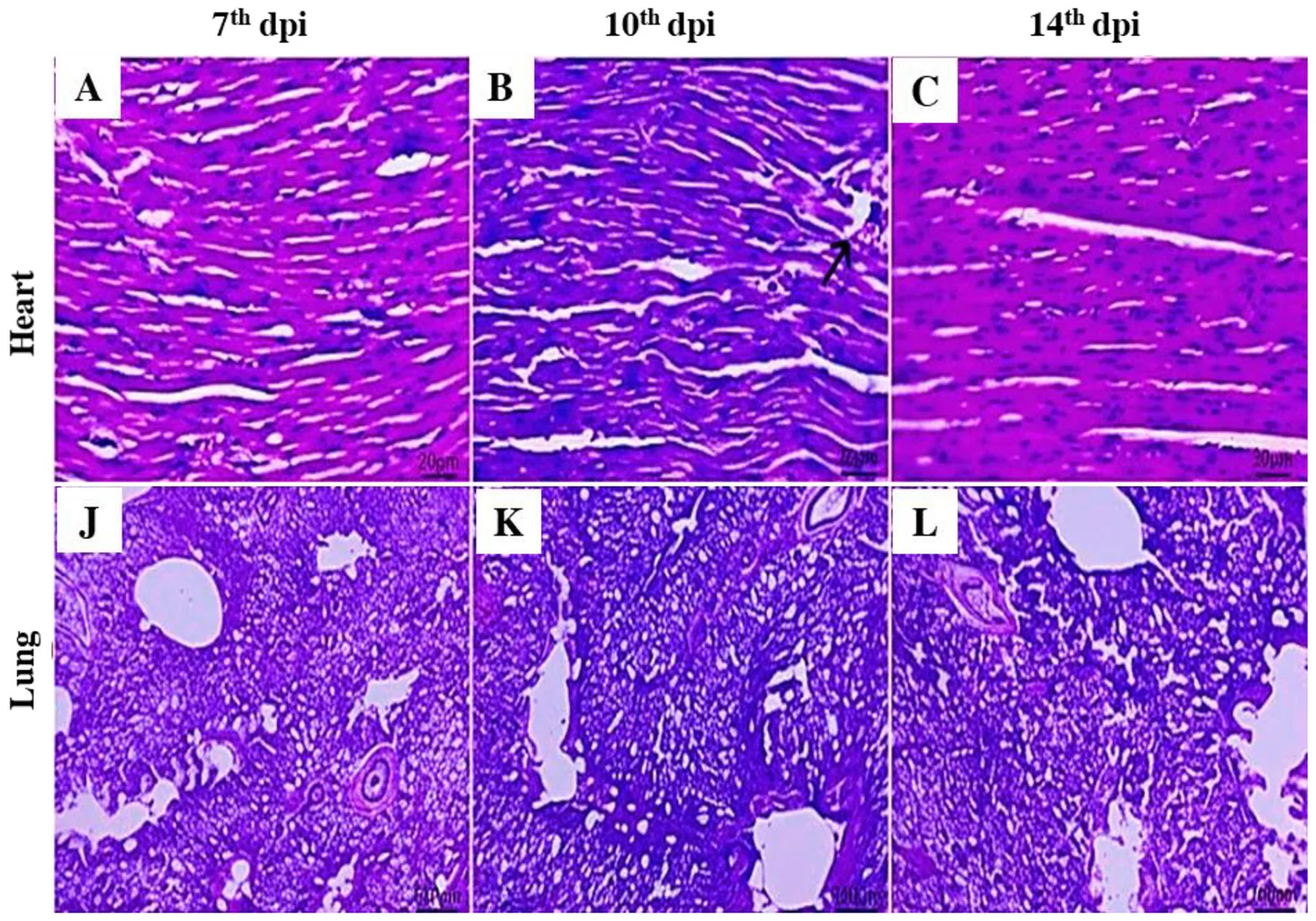
Photomicrograph of hematoxylin and eosin-stained sections from quails contacted with chickens experimentally infected with *Chlamydophila psittaci* showing heart tissues **(A–C)**. **(A)**: normal histological structures of heart muscles and interstitial tissues at 7th dpi. **(B)**: apparent normal cardiomyocytes beside mild dilated interstitial blood vessels (arrow) at 10th dpi. **(C)**: preserved histological architectures of heart muscles and interstitial tissues at 14th dpi, scale bar (20 μm). Pulmonary tissues **(J–L)**. **(J)**: apparent normal pulmonary tissues with mild perivascular edema at 7th dpi. **(K, L)**: apparent normal pulmonary architectures with mild dilated some pulmonary blood vessels at 10th dpi, 14th dpi. Scale bar (100 μm). dpi, days post-infection.

**Figure 8:**
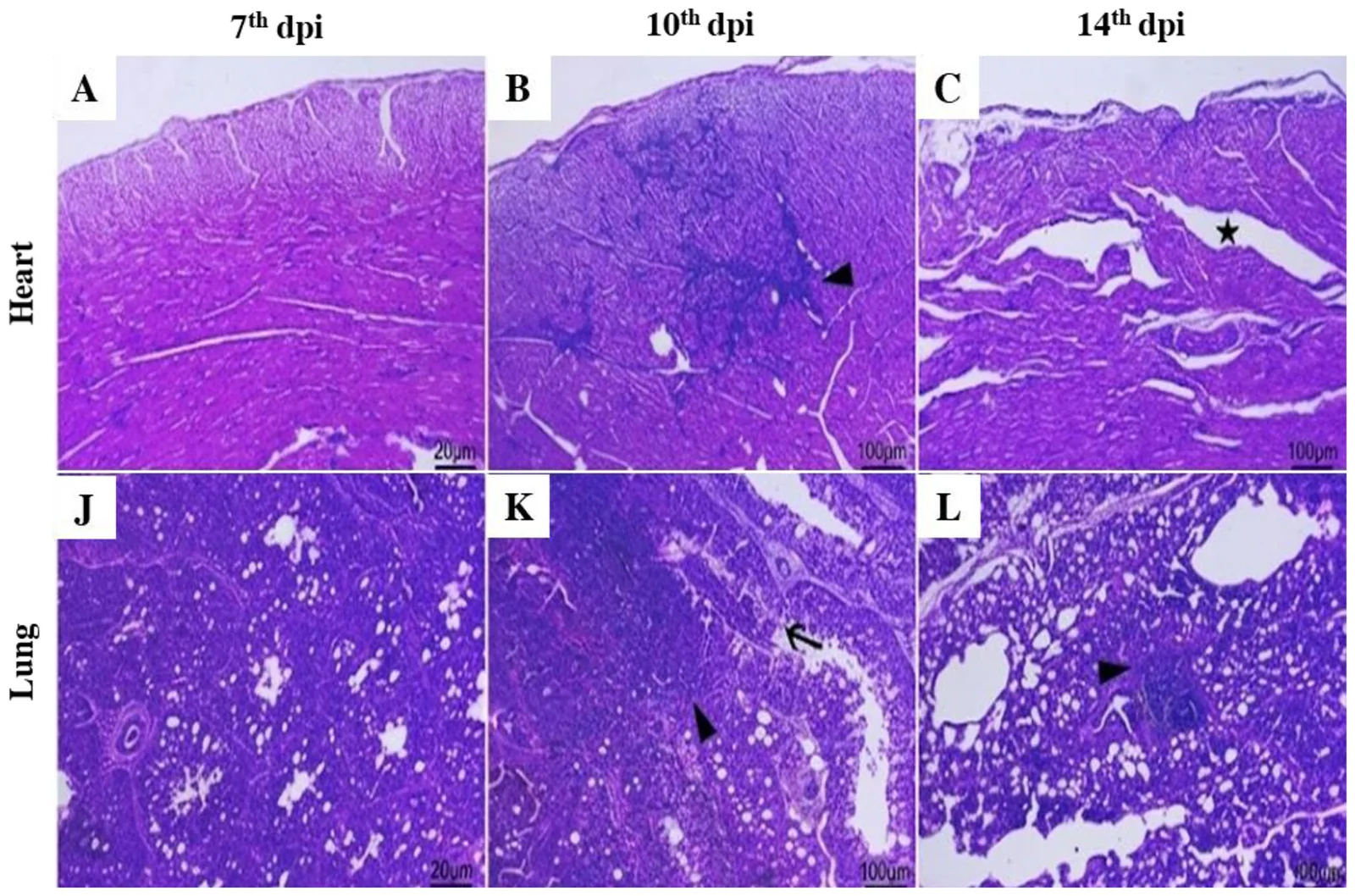
Photomicrograph of hematoxylin and eosin-stained sections from ducks contacted with chickens experimentally infected with *Chlamydophila psittaci* showing heart tissues **(A–C)**. **(A)**: normal histological structures of cardiomyocytes and interstitial tissue at 7th dpi. **(B)**: thickened pericardium, and fibrous tissues infiltrated by inflammatory cells in between degenerated cardiomyocytes at 10th dpi. **(C)**: apparent normal cardiac muscle fibers with mild thickening of pericardium, perivascular lymphocyte infiltrates, and mild interstitial edema at 14th dpi, scale bar (20 μm). Pulmonary tissues **(J–L)**. **(J)**: normal histology of pulmonary tissues at 7th dpi. **(K)**: pneumonic area represented by stenosis of air capillaries by inflammatory cells beside congestion, perivascular exudates, and exudation within some intrabronchial lumina at 10th dpi. **(L)**: normal histology of pulmonary tissue with focal aggregates of lymphoid elements at peribronchial tissues at 14th dpi, scale bar (100 μm). dpi, days post-infection.

The heart exhibited mild to moderate histopathological alterations in most infected birds, including pericardial thickening, vascular congestion, interstitial edema, inflammatory cell infiltration, and occasional hyaline degeneration of cardiomyocytes. These changes were most evident in chickens, turkeys, and ducks, whereas pigeons and quails showed minimal or no significant cardiac alterations. Cardiac lesions followed a biphasic temporal pattern, with the greatest severity observed at 10 dpi, followed by substantial resolution by 14 dpi, indicating the reversible nature of the inflammatory response in cardiac tissues (Figures 4–8).

The lungs generally showed mild and transient histopathological alterations, characterized by vascular congestion, perivascular edema, inflammatory cell infiltration, and occasional bronchial or air-capillary exudation. Ducks developed the most severe pulmonary pathology at 10 dpi, including focal pneumonia, whereas the other examined species exhibited only mild respiratory changes. Pulmonary lesions resolved rapidly, with lung architecture approaching normality by 14 dpi. Only minor residual lymphoid aggregates or exudates were observed in some birds at this time point (Figures 4–8).

The liver was among the most consistently affected organs across the examined species. The principal hepatic lesions included hepatocellular degeneration, vascular congestion, inflammatory cell infiltration, Kupffer cell activation, and focal necrosis. Hepatic changes were most pronounced between 7 and 10 dpi. Pigeons and ducks developed the most severe hepatic lesions, whereas chickens and turkeys exhibited moderate alterations and quails showed only mild changes. By 14 dpi, hepatic architecture was progressively restored, although mild residual inflammatory infiltrates persisted in some species, indicating substantial tissue repair (Supplementary Figures S4–S8).

Renal lesions varied among bird species and were characterized primarily by tubular epithelial degeneration and necrosis, dilatation of tubular lumina, vascular congestion, and interstitial inflammatory infiltration. The most pronounced renal alterations occurred in ducks, pigeons, and turkeys, while chickens exhibited moderate lesions and quails maintained nearly normal renal morphology throughout the experimental period.

Renal lesions followed a transient course, reaching maximal severity at 10 dpi and subsequently showing significant recovery by 14 dpi, consistent with a self-limiting inflammatory response. Most renal tissues demonstrated substantial restoration of their normal morphology by the end of the observation period (Supplementary Figures S4–S8).

The spleen remained largely unaffected throughout the experimental period, with its histological architecture generally preserved and no substantial pathological alterations detected compared with the other examined organs. This finding was consistent with the spleen being the least affected organ during the course of infection.

Overall, the histopathological findings demonstrated that infection induced predominantly mild to moderate, reversible lesions involving multiple organs, with marked differences in lesion severity among bird species. Pigeons and ducks exhibited the greatest overall tissue damage, particularly in the liver and kidneys, whereas quails were the least affected species, maintaining relatively normal renal morphology and showing only mild alterations in other organs.

Across the tissues examined, the predominant pattern was progressive lesion development during the early and intermediate stages of infection, followed by substantial resolution and restoration of tissue architecture by 14 dpi.

### Bacterial shedding using qRT-PCR

As shown in Figure 9 and Supplementary Figure S9, the cycle threshold (Ct) values for all four avian species were minimal around the 10th dpi, indicating the peak phase of maximum bacterial proliferation. Quails and ducks showed relatively stable Ct values across the study period with no clear reduction. Turkeys exhibited the most orderly upward trend (increasing Ct values over time), suggesting progressive clearance of the infection.

**Figure 9:**
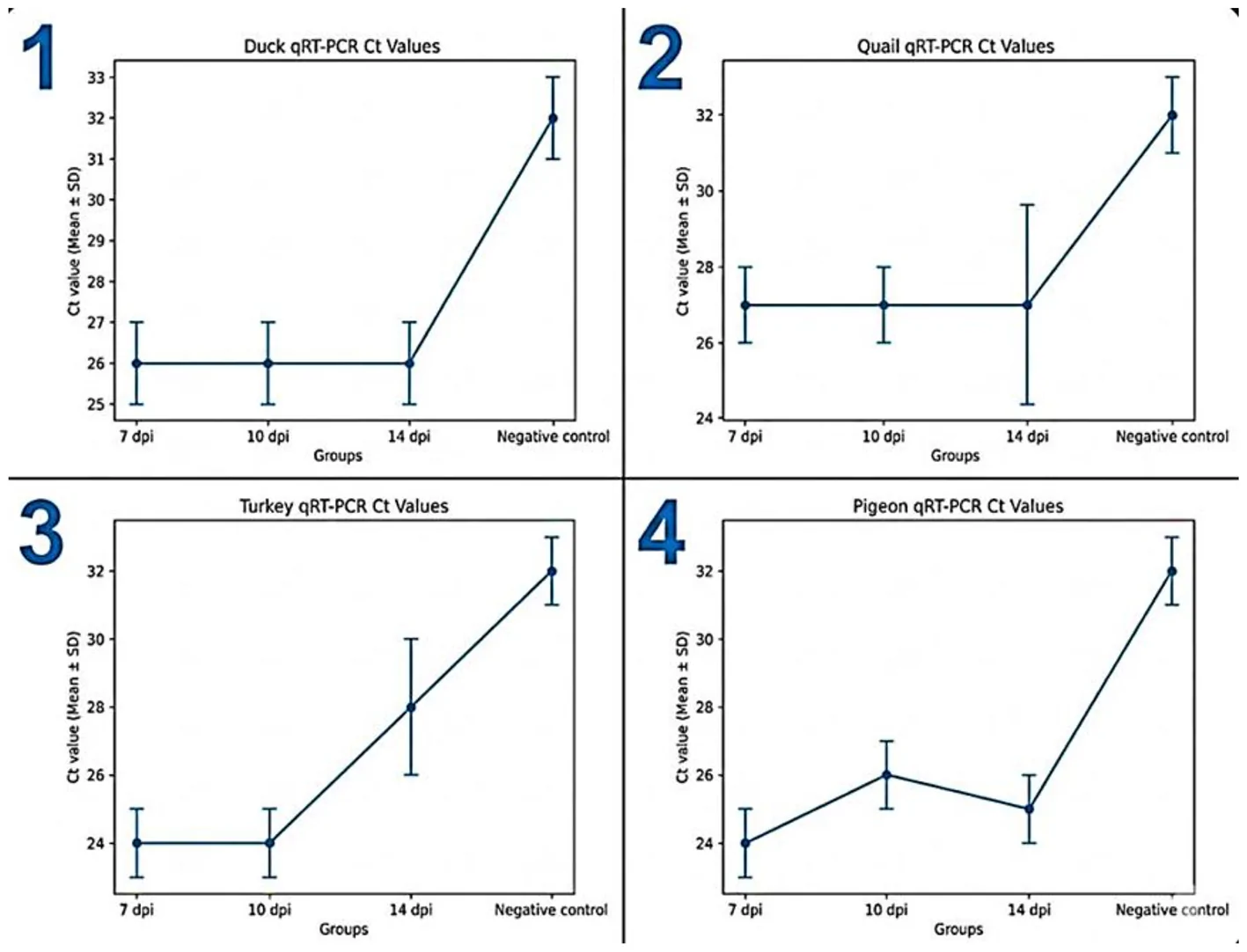
Ct values of qRT-PCR detection of *Chlamydophila psittaci* in different avian species (quails, ducks, pigeons, and turkeys) contacted with chickens experimentally infected with *Chlamydophila psittaci* at 7, 10, and 14 days post-infection (dpi). Data are expressed as mean ± SD of three replicates. Negative control reactions showed Ct values >30. Negative control values (Ct > 30) are presented as a reference baseline and do not represent a 14-days post-infection time point. Ct value, cycle threshold value. **(1)** duck qRT-PCR Ct values; **(2)** quail qRT-PCR Ct values; **(3)** turkey qRT-PCR Ct values; and **(4)** pigeon qRT-PCR Ct values.

In contrast, pigeons showed more variable Ct values, reflecting greater inter-individual variation in bacterial shedding patterns. These findings reflect species-related differences in susceptibility, immune response, and bacterial persistence.

## Discussion

Avian chlamydiosis, caused by *C. psittaci*, is a zoonotic disease of major veterinary and public health importance (14, 38). It affects a wide range of avian species, including poultry, pet birds, and wild birds, leading to clinical outcomes that range from subclinical infections to severe respiratory and systemic illness (39). Infected birds can shed the pathogen intermittently through feces, respiratory secretions, and feather dust, posing a significant risk of transmission to humans, where it causes psittacosis, a potentially serious respiratory disease (40).

Beyond its zoonotic threat, avian chlamydiosis has substantial economic implications due to reduced productivity, trade restrictions, and increased costs of control and biosecurity measures (20). Therefore, understanding and managing this disease is crucial for safeguarding both animal health and human health under the One Health framework (41, 42).

In the current study, no mortality was recorded during the 35-day experimental period in all the infected avian species (chickens, ducks, turkeys, quails, and pigeons). This observation is comparable to that reported by Hegazy et al. (9), who experimentally challenged chickens with *C. psittaci*. The present study also demonstrated the successful inter-species transmission of *C. psittaci* from experimentally infected chickens to other avian species under co-housing conditions. However, infection severity varied among the species examined, with turkeys showing more pronounced clinical signs, greater bacterial shedding, and more severe histopathological lesions than ducks, pigeons, and quails.

The clinical signs detected in this study were as follows: in chickens, signs appeared at 3–5 dpi and included depression, weakness, ruffled feathers, diarrhea (of varying colors), and respiratory issues (gasping of air, conjunctivitis, nasal discharge, sneezing, coughing). Furthermore, ducks exhibited signs of nervousness (paddling) at 14 dpi. Post-contact (3–5 days), turkeys were most affected (13 birds affected out of 15), chickens, ducks, and pigeons showed moderate signs, and quails showed mild signs. These findings are parallel to those reported by Hegazy et al. (9) in chickens; Ciftçi et al. (43) in turkeys; Vorimore et al. (44) in ducks; Hegazy et al. (35) in quails; and Sachse et al. (45) in pigeons. Furthermore, in the current study, turkeys exhibited the highest severity, with nearly all birds (11–15 of 15) displaying clinical signs and major lesions, indicating they are the most susceptible to psittacosis.

In addition, in the current study, chickens, ducks, and pigeons had moderate severity (6–10 birds affected out of 15), placing them in the intermediate category. Quails exhibited the lowest scores (1–5 birds affected out of 15), indicating a modest level of susceptibility. These findings concur with those reported by Hegazy et al. (35) and Zhou et al. (46), who confirmed that psittacosis presents with similar clinical signs across avian species.

Vanrompay (47) also noted that the clinical signs of avian psittacosis were similar across avian species. For example, in pigeons, conjunctivitis, blepharitis, and rhinitis are common and are occasionally accompanied by transient ataxia. Disease severity in turkeys varies, with signs including conjunctivitis, respiratory problems, diarrhea, and decreased egg production; mortality rates range from minimal to high, and some birds may show reduced weight gain without overt signs.

A rare syndrome characterized by inflammation of the nasal glands and periocular edema, devoid of respiratory symptoms or mortality, has been documented in turkeys (47). In addition, ducks exhibited tremors and gait abnormalities, although many infections are subclinical; likewise, peafowl often develop diarrhea and respiratory signs, while rheas and raptors may experience sudden death following weight loss (47). However, clinical signs are rare in chickens, though experimental infections have induced respiratory symptoms and conjunctivitis (47).

Based on the current data, PM and lesion scoring revealed diverse lesions, including hepatomegaly, splenomegaly, renal and pulmonary congestion, enteritis, and air sacculitis of varying severity (foamy to fibrinous). Lesion severity was highest in turkeys, moderate to moderately severe in ducks and pigeons, moderate in chickens, and mild to moderate in quails. The PM lesions recorded were parallel to those reported by Hegazy et al. (35) and Zhou et al. (46) across different avian species, with variable degrees of severity, ranging from mild to moderate to severe.

From the current data, the infected birds (chickens, turkeys, ducks, quails, and pigeons) showed different degrees (mild, moderate, and/ or severe) of histopathological changes in the examined organs, such as heart sections at 7th dpi, which showed normal cardiomyocytes with dilated interstitial vessels and pericardial inflammation. Mild myofiber hyaline degeneration was noted at 10th dpi, with normal morphology restored, clarifying the observed change in lesion severity by time, particularly the improvement at later stages (14th dpi).

Liver samples exhibited focal inflammatory infiltrates and vascular dilation at 7th dpi, hepatocyte degeneration and vascular congestion at 10th dpi, and normal hepatic architecture. This explains the observed change in lesion severity by time, particularly the improvement at later stages (14th dpi). Kidney sections showed normal structures at 7th dpi; however, degenerative tubular changes, necrosis, luminal dilation, and vascular congestion were observed at 10th dpi and 14th dpi. Lung tissues maintained normal pulmonary architecture with vascular congestion and perivascular edema at 7th dpi, normal morphology at 10th dpi, and residual bronchial exudates at 14th dpi. Spleen histology remained consistent across 7th, 10th, and 14th dpi, with intact white and red pulp structures.

Based on qRT-PCR, bacterial shedding of *C. psittaci* decreased over time in all tested avian species (chickens, turkeys, ducks, quails, and pigeons). These data agreed with those reported by Hegazy et al. (9), who found that bacterial shedding diminished over time in chickens experimentally infected with *C. psittaci*. The current results (Figure 9) showed lower Ct values at 10th dpi, indicating a higher bacterial load during this stage of infection. This trend is consistent with previous experimental studies reporting peak bacterial replication during the early-to-mid phase of infection in poultry. Similar Ct value patterns have been reported in experimental infections, in which bacterial shedding increased during the acute phase and gradually declined as the host immune response developed (9).

In the present study, macroscopic and microscopic lesion scoring throughout the experimental period showed that clinical signs, PM lesions, histopathological alterations, and bacterial shedding decreased in severity over time, with a noticeable improvement in the tissues. Bowman et al. (48) confirmed that in the chronic stage of *C. psittaci* infection, birds often exhibit mild or unapparent clinical signs, which makes detection difficult. Despite the absence of overt illness, these birds can intermittently shed the pathogen through feces and respiratory secretions, especially under stress conditions such as transportation, overcrowding, or concurrent infections (5).

Several biological factors may explain the observed differences in susceptibility among bird species. Variations in host immune responses could influence different avian species' ability to control bacterial replication. In addition, differences in cellular receptor expression and epithelial cell susceptibility may affect bacterial attachment, entry, and replication within host tissues. Such species-specific physiological and immunological factors may therefore contribute to the higher susceptibility observed in turkeys.

This carrier state poses a significant risk, as chronically infected birds act as reservoirs for continuous transmission within and between flocks and to humans (49). The persistence of *C. psittaci* in carrier birds not only hampers eradication efforts but also contributes to recurrent outbreaks, leading to economic losses in the poultry industry and serious zoonotic threats to public health (50).

Finally, molecular and immunological analyses to assess host immune responses and pathogen–host interactions at the molecular level, to understand mechanisms underlying variable susceptibility, should be conducted in parallel with vaccine development to explore the potential for effective vaccination strategies or immunomodulatory interventions to mitigate transmission and clinical impact, to control and prevent this zoonotic problem.

The incidence of *C. psittaci* transmission among domestic birds is high, particularly in turkeys, posing a potential threat to human health in the study area. Therefore, further zoonotic risk assessments are recommended to evaluate the risk of transmission from various avian species to humans, particularly among poultry-farm workers, live-bird-market workers, and pet-bird handlers.

## Conclusion

This study demonstrated the efficient inter-species transmission of *C. psittaci* from experimentally infected chickens to several avian species under co-housing conditions. Turkeys showed the highest susceptibility, with more pronounced clinical signs, higher bacterial loads, and more severe histopathological lesions than ducks, pigeons, and quails. The findings highlight the important role of chickens as a potential reservoir for *C. psittaci* dissemination in mixed-species poultry systems. These results emphasize the need for continuous monitoring of bacterial shedding and improved biosecurity strategies to reduce transmission risk among poultry species. Strict biosecurity measures are essential on mixed-species poultry farms. Special emphasis must be placed on biosecurity practices specific to turkeys, given their heightened vulnerability to infection. Routine molecular surveillance using qRT-PCR, along with improved species-specific separation, is recommended to minimize the spread of *C. psittaci* and protect poultry health.

## Data Availability

The raw data supporting the conclusions of this article will be made available by the authors, without undue reservation.
