# Supplementary material for: Inter-species transmission of Chlamydophila psittaci from broiler chickens to ducks, turkeys, pigeons, and quails: an experimental study
Source: Front Vet Sci. 2026 Oct 22;13:1870801. doi: 10.3389/fvets.2026.1870801 (PMC13735094; doi:10.3389/fvets.2026.1870801)
Supplement: Supplementary file 1 [file Data_Sheet_1.docx]

***Supplementary Materials***

**Inter-species transmission of *Chlamydophila psittaci* from broiler chickens to ducks, turkeys, pigeons, and quails: an experimental study**

Ahmed M. E. Hegazy, Amira M. Morsy, Heba M. Salem, Jawza A. Almutairi, Hadil Alkathiry, Yasser S. Mostafa, Ibrahim Mufadhi M Alanazi, Suad Hamdan Almasoudi, Khaled A. El-Tarabily*, and Hala M.N. Tolba

Corresponding author: ktarabily@uaeu.ac.ae (Khaled A. El-Tarabily)

## **FIGURE S1**

## Prevalence of clinical signs among different experimental groups (infected chickens with *Chlamydophila psittaci*, contacted birds (ducks, turkeys, quails, and pigeons), and negative control chickens). Turkeys showed the most severe clinical signs, while chickens, ducks, and pigeons showed moderate signs, but quails showed only mild signs.

**FIGURE S2**

Prevalence and distribution of A: liver lesions; B: spleen lesions; C: air sacculitis; D: enteritis across poultry species.

**FIGURE S3**

Photomicrographs of hematoxylin and eosin-stained sections from the chickens' negative control group showing heart tissues (A-C); A: with normal striated branched cardiac muscle fibers and interstitial tissues. at 7^th^ dpi, 10^th^ dpi, and at 14^th^ dpi, scale bar (20 µm); liver tissues (D-F) with normal histology of hepatic acini, central veins, sinusoids, Kupffer cells, and portal areas at 7^th^ dpi, 10^th^ dpi, and at 14^th^ dpi, scale bar (20 µm); kidney sections (G-I); with normal histology of renal corpuscles, renal tubules and stromal structures at 7^th^ dpi, 10^th^ dpi, and at 14^th^ dpi, scale bar (20 µm); pulmonary tissues (J-L) with normal morphological structures of bronchi, bronchioles, air vesicles, and vascular tissues at 7^th^ dpi, 10^th^ dpi, and at 14^th^ dpi, scale bar (100 µm). dpi, days post-infection.

**FIGURE S4**

Photomicrograph of hematoxylin and eosin-stained sections from chickens experimentally infected with *Chlamydophila psittaci* showing liver tissues (D-F); D: focal areas of inflammatory cells infiltrates (arrowhead) and dilated vasculatures at 7^th^ dpi. E: degenerated some hepatocytes (arrowhead) and congested hepatic blood vessels (arrow) in the group at 10^th^ dpi. F: normal morphology of hepatic acini, and central veins at 14^th^ dpi, scale bar (20 µm; kidney sections (G-I); G: normal morphology of renal tubules and glomerular corpuscles at 7^th^ dpi. H: degenerative changes in some tubular epithelium, necrotic, with a few tubules, dilated renal tubular lumina (star) at 10^th^ dpi. I: degenerative changes of some renal tubules at 14^th^ dpi, scale bar (20 µm). dpi, days post-infection.

**FIGURE S5**

Photomicrograph of hematoxylin and eosin-stained sections from turkeys contacted with chickens experimentally infected with *Chlamydophila psittaci* showing liver tissues (D-F); D: degeneration of some hepatocytes (arrow), focal area of interstitial inflammatory cells infiltrates (arrowhead), and hypertrophy of Kupffer cells at 7^th^ dpi. E: focal areas of inflammatory cell infiltration (arrowhead) and dilated hepatic vasculatures at the 10^th^ dpi. F: Normal histological architectures of hepatic acini, central vein, and sinusoids at 14^th^ dpi, scale bar (20 µm); kidney sections (G-I); G: degenerated and necrotic renal tubular epithelium (arrow) at 7^th^ dpi. H: a few necrotic tubules (arrow) at 10^th^ dpi. I: normal histology of kidney corpuscles, renal tubules, and stromal structures at 14^th^ dpi, scale bar (20 µm). dpi, days post-infection.

**FIGURE S6**

Photomicrograph of hematoxylin and eosin-stained sections from pigeon contacted with chickens experimentally infected with *Chlamydophila psittaci* showing liver tissues (D-F); D: degenerated hepatocytes, congestion of hepatic blood vessel (arrow), perivascular inflammatory cells infiltrate (arrowhead), and focal area of necrotic hepatocytes replaced by inflammatory cells at 7^th^ dpi. E: congested vasculature (arrow), marked degenerative changes in almost all hepatic parenchyma, primarily fatty change, besides multifocal areas of hepatic necrosis surrounded by an inflammatory line (arrowhead) at 10^th^ dpi. F: apparent normal histological architectures of hepatic acini, hepatic vasculatures with interstitial lymphocyte infiltrates (arrowhead) at 14^th^ dpi, scale bar (100 µm); kidney sections (G-I); G: necrotic some renal tubular epithelium, dilated some renal tubular lumina (star) and focal area of interstitial inflammatory cells infiltrates at 7^th^ dpi. H: some necrotic tubules (arrowhead), and congestion of interstitial blood vessels at 10^th^ dpi. I: normal histology of kidney corpuscles, renal tubules and stromal features at 14^th^ dpi, scale bar (20 µm). “BALT” (arrowheads) at 10^th^ dpi. L: normal histological structures of bronchi, bronchioles, air vesicles, and vascular tissues (arrowhead) at 14^th^ dpi, scale bar (100 µm). dpi, days post-infection.

**FIGURE S7**

Photomicrograph of hematoxylin and eosin-stained sections from quails contacted with chickens experimentally infected with *Chlamydophila psittaci* showing liver tissues (D-F); D, E: apparent normal histological structures of hepatocytes with congested hepatic blood vessels (arrow) at 7^th^ dpi, at 10^th^ dpi. F: apparent normal histological structures of most hepatic parenchyma with mild degeneration within some hepatocytes at 14^th^ dpi, scale bar (100 µm); kidney sections (G-I); G, H, I: normal morphological structures of renal tubules, glomerular corpuscles and stromal tissues at 7^th^ dpi,10^th^ dpi, 14^th^ dpi, scale bar (100 µm). dpi, days post-infection.

**FIGURE S8**

Photomicrograph of hematoxylin and eosin-stained sections from ducks contacted with chickens experimentally infected with *Chlamydophila psittaci* showing liver tissues (D-F); D: vacuolated most hepatic parenchyma at 7^th^ dpi. E: degenerated hepatic parenchyma, focal areas of inflammatory cell infiltration, and areas of coagulative necrosis surrounded by inflammatory cell infiltrates at 10^th^ dpi. F: apparent normal architectures of hepatic acini with some congested hepatic blood vessels at 14^th^ dpi, scale bar (100 µm); kidney sections (G-I); G: normal histological features of kidney corpuscles, renal tubules, and stromal structures at 7^th^ dpi. H: numerous necrotic tubules and interstitial inflammatory cells infiltrate at 10^th^ dpi. I: a few numbers of inflammatory cells infiltrate in between apparent normal kidney parenchyma at 14^th^ dpi, scale bar (20 µm). dpi, days post-infection.

**FIGURE S9**

Amplification curve for oropharyngeal swabs in chickens at 7^th^ dpi of experimental infection with *Chlamydophila psittaci*. dpi, days post-infection.
